# Potential of Melt Electrowritten Scaffolds Seeded with Meniscus Cells and Mesenchymal Stromal Cells

**DOI:** 10.3390/ijms222011200

**Published:** 2021-10-18

**Authors:** Jasmijn V. Korpershoek, Mylène de Ruijter, Bastiaan F. Terhaard, Michella H. Hagmeijer, Daniël B.F. Saris, Miguel Castilho, Jos Malda, Lucienne A. Vonk

**Affiliations:** 1 Department of Orthopaedics, University Medical Center Utrecht, 3584 CX Utrecht, The Netherlands; j.v.korpershoek-3@umcutrecht.nl (J.V.K.); M.deRuijter@umcutrecht.nl (M.d.R.); bastiaanterhaard@gmail.com (B.F.T.); M.H.Hagmeijer-2@umcutrecht.nl (M.H.H.); d.saris@umcutrecht.nl (D.B.F.S.); M.DiasCastilho@umcutrecht.nl (M.C.); 2 Department of Orthopedic Surgery and Sports Medicine, Mayo Clinics, Rochester, MN 55905, USA; 3 Department of Reconstructive Medicine, University of Twente, 7522 NB Enschede, The Netherlands; 4 Department of Biomedical Engineering, Eindhoven University of Technology, 5612 AZ Eindhoven, The Netherlands; 5 Department of Clinical Sciences, Faculty of Veterinary Medicine, Utrecht University, 3584 CS Utrecht, The Netherlands

**Keywords:** meniscus, collagen meniscus implant^®^, melt electrowriting, tissue-engineering, biofabrication, meniscus injury, clinical translation, meniscectomy18

## Abstract

Meniscus injury and meniscectomy are strongly related to osteoarthritis, thus there is a clinical need for meniscus replacement. The purpose of this study is to create a meniscus scaffold with micro-scale circumferential and radial fibres suitable for a one-stage cell-based treatment. Poly-caprolactone-based scaffolds with three different architectures were made using melt electrowriting (MEW) technology and their in vitro performance was compared with scaffolds made using fused-deposition modelling (FDM) and with the clinically used Collagen Meniscus Implants^®^ (CMI^®^). The scaffolds were seeded with meniscus and mesenchymal stromal cells (MSCs) in fibrin gel and cultured for 28 d. A basal level of proteoglycan production was demonstrated in MEW scaffolds, the CMI^®^, and fibrin gel control, yet within the FDM scaffolds less proteoglycan production was observed. Compressive properties were assessed under uniaxial confined compression after 1 and 28 d of culture. The MEW scaffolds showed a higher Young’s modulus when compared to the CMI^®^ scaffolds and a higher yield point compared to FDM scaffolds. This study demonstrates the feasibility of creating a wedge-shaped meniscus scaffold with MEW using medical-grade materials and seeding the scaffold with a clinically-feasible cell number and -type for potential translation as a one-stage treatment.

## 1. Introduction

The human meniscus is a fibrocartilaginous tissue in the knee that shows a distinct architecture with an inner zone, composed of hyaline cartilage-like tissue, and an outer zone with a more fibrous phenotype [1,2]. It plays a crucial role in load transmission in the knee due to an organized network of circumferential and radial collagen fibres [1,3,4]. Meniscus injury is highly disabling and affects young, active patients, as well as the elderly. The regenerative capacity of the meniscus is limited to the vascular zone and declines with aging. Therefore, successful surgical repair of meniscal tears is limited to the vascularized region and to young patients [5,6]. Because roughly 66% of all meniscus tears are irreparable [7], treatment often involves meniscectomy, i.e., the removal of the damaged part of the meniscus. Meniscectomy relieves symptoms in the short-term, but is related to a high risk of developing osteoarthritis due to loss of contact area between the long bones and altered load bearing [8,9,10]. Current strategies for replacement of the meniscus have important drawbacks. Transplantation of meniscus allografts is costly and highly regulated in the European Union[11]. It requires complex logistics as donor availability is limited, and high-level evidence on long-term effectiveness is lacking [12,13]. The Collagen Meniscus Implant (CMI^®^; Stryker, Kalamazoo, MI, USA), a clinically available implant composed of bovine type 1 collagen, offers short term clinical improvement, yet tissue deposition is limited in the long-term [14]. Moreover, the CMI^®^ does not account for the zonal organization and direction of collagen fibres in the meniscus. The clinical need for a mechanically competent meniscus implant is therefore unmet. Ideally, such implant should allow for sufficient dampening and load transfer while being able to remodel to the joint in vivo. In order to improve performance in the long-term and reactiveness to the joint environment, it should be biocompatible with an optimal pore size and pore interconnectivity to achieve cell infiltration and tissue ingrowth [15]. Pre-seeding a scaffold with cells could stimulate tissue formation and thereby enhance the long-term performance of a meniscus scaffold.

A potential solution to the limited mechanical properties of current implants, such as the CMI^®^ or ACTIfit (Orteq^®^ Sports Medicine Ltd., New York, NY, USA), could lie in mimicking the collagen fibre architecture of native meniscus tissue. Recent developments on additive manufacturing technologies, or more specifically, fibre deposition technologies seem promising for mimicking the complexity at native tissue resolution. Recent applications of such technologies for the fabrication of meniscus scaffolds focus on achieving a strong fused deposition modelling (FDM) polymeric framework that can be combined with previously proven hydrogel biomaterials and cells and other bioactive moieties [16,17]. Polymeric fibres that are produced by FDM (fibre diameter within the hundreds of micrometer scale) are generally stiffer as compared to thinner sub-micrometer scale fibres produced by other fibre deposition technologies such as melt electrowriting (MEW) [18]. Limitations of using a stiff supporting framework with large fibre diameters, such as done with FDM, include limited load transfer to seeded cells which consequently compromise their mechano-regulated differentiation and neo-tissue deposition. Additionally, the polymeric fibres resulting from FDM may lead to damage on the opposing articulating cartilage surfaces due their size and stiffness. Solution electrospinning can mimic the (nano-sized) fibres of native meniscus tissue, but this technology generally uses toxic solvents and does not allow controlled fibre deposition, necessitating the addition of thicker FDM-based support fibres to obtain the aligned fibre architecture [19]. For other tissues such as articular cartilage and heart muscle, controlled and aligned fibres were previously deposited using MEW to mechanically reinforce cell-laden hydrogels [18,20]. Next to this reinforcing effect, MEW fibres are made using of medical grade polymers, can be deposited with high reproducibility, allow for sufficient pore interconnectivity, and have a less rough and/or stiff surface as compared to FDM fibres due to the micro-scale of the fibres. Therefore, MEW provides potential to recapitulate the fibre architecture of native meniscus tissue, while allowing space for the cells to produce meniscus-like tissue. Such MEW scaffolds can then be used for testing or potentially implantation purposes. 

In order to facilitate clinical translation of a pre-seeded scaffold, the number of autologous cells should not exceed the number that can be harvested during a single surgical procedure. A sufficient number of cells/stimuli for tissue formation can be achieved by combining recycled autologous meniscus cells isolated from the meniscectomized tissue with allogeneic mesenchymal stromal cells (MSCs). The feasibility of using these cell combinations was already shown in a human clinical trial for cartilage defects [21], and in an in vitro experiment for the meniscus [22]. Using a combination of off-the-shelf allogeneic MSC and autologous meniscus cells that are harvested during the surgery allows for implantation in a one-stage procedure, thus limiting patient burden and costs of treatment [23,24].

In this study, we demonstrate feasibility of fabrication of a wedge-shaped meniscus scaffold with circumferential and radial fibres, made from medical grade materials with MEW. We used a combination of meniscus cells and MSCs to seed the scaffold with a clinically feasible cell-source and number for one-stage meniscus replacement. Compressive properties were assessed under confined uniaxial loading and proteoglycan production was assessed after 28 d of culture. 

## 2. Results

### 2.1. Scaffold Fabrication

**Figure 1:**
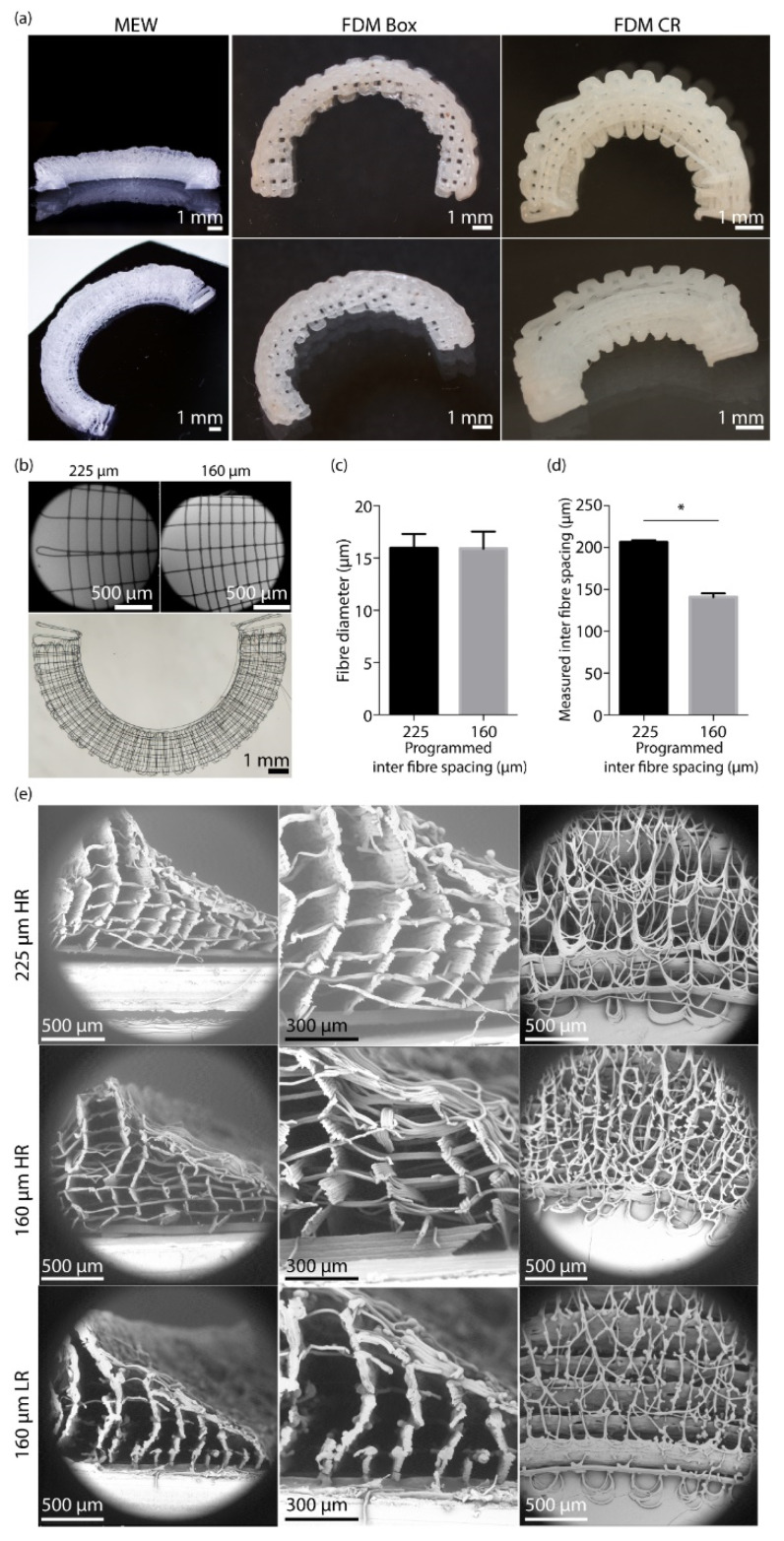
Printability of circumferential and radial melt electrowritten fibres to achieve a wedge shaped meniscus. (**a**) Macroscopic image of Melt electrowritten (MEW) scaffold, Fused deposition Modelling ( FDM) scaffold with an inner boxed-shaped (Box) architecture, and FDM scaffold with an inner circumferential/radial (CR) architecture. (**b**) Top view of a single layer of circumferential and radial fibres. (**c**) Fibre diameter of fibres for both inter fibre spacings (*n* = 3 per group). (**d**) Measured inter fibre spacing for both programmed inter fibre spacings (*n* = 3 per group). (**e**) Scanning electron microscopy images of scaffolds with both inter fibre spacings and the different ratios of circumferential and radial fibres. * = *p* < 0.05. Abbreviations; HR: high ratio of radial fibres, LR: low ratio of radial fibres.

MEW scaffolds macroscopically showed the native meniscus wedge-like shape (Figure 1A). The two different FDM scaffolds, boxes-shaped (Box) and circumferential/radial-shaped (CR), macroscopically showed the wedge architecture, yet in a lower resolution (Figure 1A). A distinction between the circumferential and radial fibres was observed upon alternating these layers for both 225 μm and 160 μm inter-fibre spacings (Figure 1B). An average fibre diameter of 15.9 ± 1.6 μm was found for the 225 μm programmed inter fibre spacing and an average fibre diameter of 15.8 ± 1.6 μm was found for the 160 μm spacing (Figure 1B). Additionally, the measured inter fibre spacing was close to the programmed line spacing and showed a high reproducibility (Figure 1C,D). On a microscopic level, the wedge shape could be observed for both the 225 μm and 160 μm fibre spacing (Figure 1E). Furthermore, the difference in circumferential and radial fibres and the variation in ratio (HR, high ratio of radial fibres; LR, low ratio of radial fibres) of these radial fibres is shown (Figure 1E).

### 2.2. Seeding and Culture of Scaffolds

**Figure 2:**
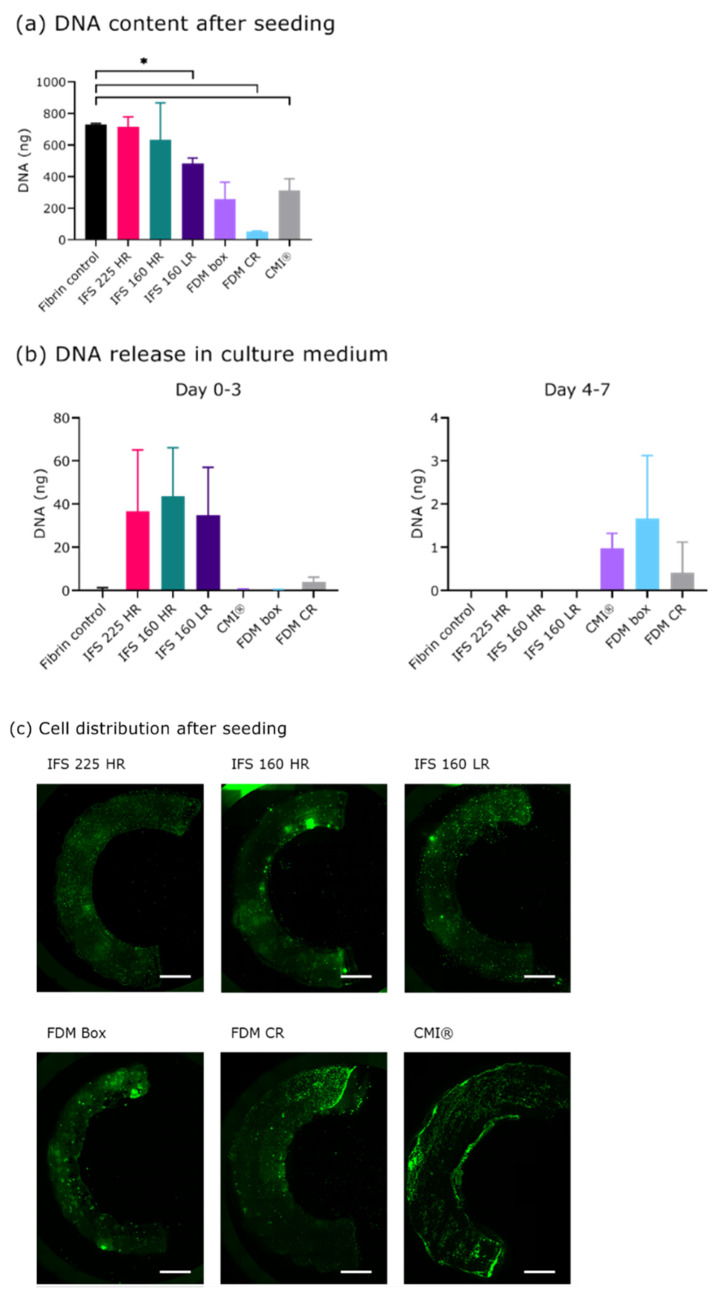
Cell seeding and DNA release into the culture medium. (**a**) DNA content of seeded scaffolds and fibrin gel controls. (**b**) DNA release in culture medium as indirect measure of cell retention in the scaffold in the first week after seeding (*n* = 3 technical replicates, 1 donor). (**c)** Cell distribution 1 day after seeding, green fluorescent dye is Calcein AM. *, *p* < 0.05. Abbreviations; 160: 160 µm, 225: 225 µm, CMI^®^: Collagen Meniscus Implant^®^, IFS: inter fibre spacing, HR: high ratio of radial fibres, LR: low ratio of radial fibres, FDM: fused deposition modelling, CR: circumferential and radial fibres, Box: box-structure. Scale bar is 2 mm.

The DNA content of seeded scaffolds was comparable between the HR MEW-scaffolds and a fibrin gel control, indicating successful seeding of these scaffolds (Figure 2A). The FDM CR and CMI^®^ contained significantly less DNA after seeding than the fibrin control. No significant differences were found between the different scaffold geometries. DNA release into the medium was minimal compared to the DNA content after seeding, indicating good retention of the cells in the scaffolds (Figure 2B). All scaffolds showed homogeneous distribution of live cells in the scaffold as shown by calcein AM staining (Figure 2C). 

### 2.3. Mechanical Properties of In Vitro Cultured Scaffolds 

**Figure 3:**
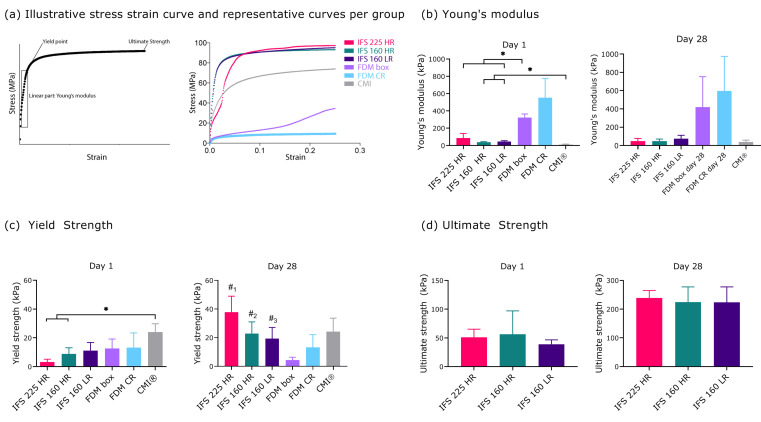
Mechanical characteristics of scaffolds seeded with co-cultured mesenchymal stromal cells and meniscus cells (80:20) in fibrin gels 1 day after seeding and after 28 d of culture (*n* = 3 donors per group, 3 technical replicates per donor)**.** (**a**) Illustrative stress strain curve and representative stress strain curves of measured groups. (**b**) Young’s Modulus (**c**) Yield Strength (**d**) Ultimate strength. *, *p* < 0.05 compared to all groups except IFS 160 HR; #_2_, *p* < 0.05 compared to all groups; #_3_, *p* < 0.05 compared to all groups except CMI^®^ and FDM CR; #_4_, *p* < 0.05 compared to FDM box and IFS 225 HR. Abbreviations; 160: 160 µm, 225: 225 µm, CMI^®^: Collagen Meniscus Implant^®^, IFS: inter fibre spacing, HR: high ratio of radial fibres, LR: low ratio of radial fibres, FDM: fused deposition modelling, CR: circumferential and radial fibres, Box: box-structure.

Mechanical properties were assessed after 1 and 28 d of culture. The stress strain curves of different groups show similar behaviour upon compressive loading, yet at different strain values (Figure 3A). At day 1, MEW scaffolds with an inter fibre spacing of 160 µm (40 ± 7 and 46 ± 11 kPa for HR and LR, respectively) showed a significantly higher compressive Young’s modulus than the CMI^®^ scaffolds (13 ± 5 kPa) (Figure 3B). The FDM Box scaffold had a significantly higher Young’s modulus than the CMI^®^ and all MEW scaffolds (Figure 3B). At day 1, the yield strength was achieved at a higher strain for the MEW scaffolds (73 ± 8 and 70 ± 11 for the IFS 160 HR and IFS 160 LR, respectively) as compared to the FDM scaffolds (23 ± 2 and 11 ± 5, for the Box and CR, respectively), indicating a larger elastic region for the MEW scaffolds (Appendix A, Figure A2). At day 28, all MEW groups showed the yield point at a higher strain as compared to the FDM groups. Yield strength was comparable between the MEW and FDM scaffolds at day 1, whereas the CMI^®^ (24 ± 5 kPa) had a higher yield strength than the IFS 225 HR (3 ± 2 kPa) and IFS 160 HR (8 ± 4 kPa) (Figure 3C). At day 28, yield strength in MEW groups had increased (Figure 3C). Ultimate strength of CMI^®^ and FDM groups was not above the higher limits of the testing set-up. Ultimate strength of MEW groups increased between day 1 and day 28 and did not differ significantly between groups (Figure 3D).

### 2.4. Scaffold Shape Fidelity 

**Figure 4:**
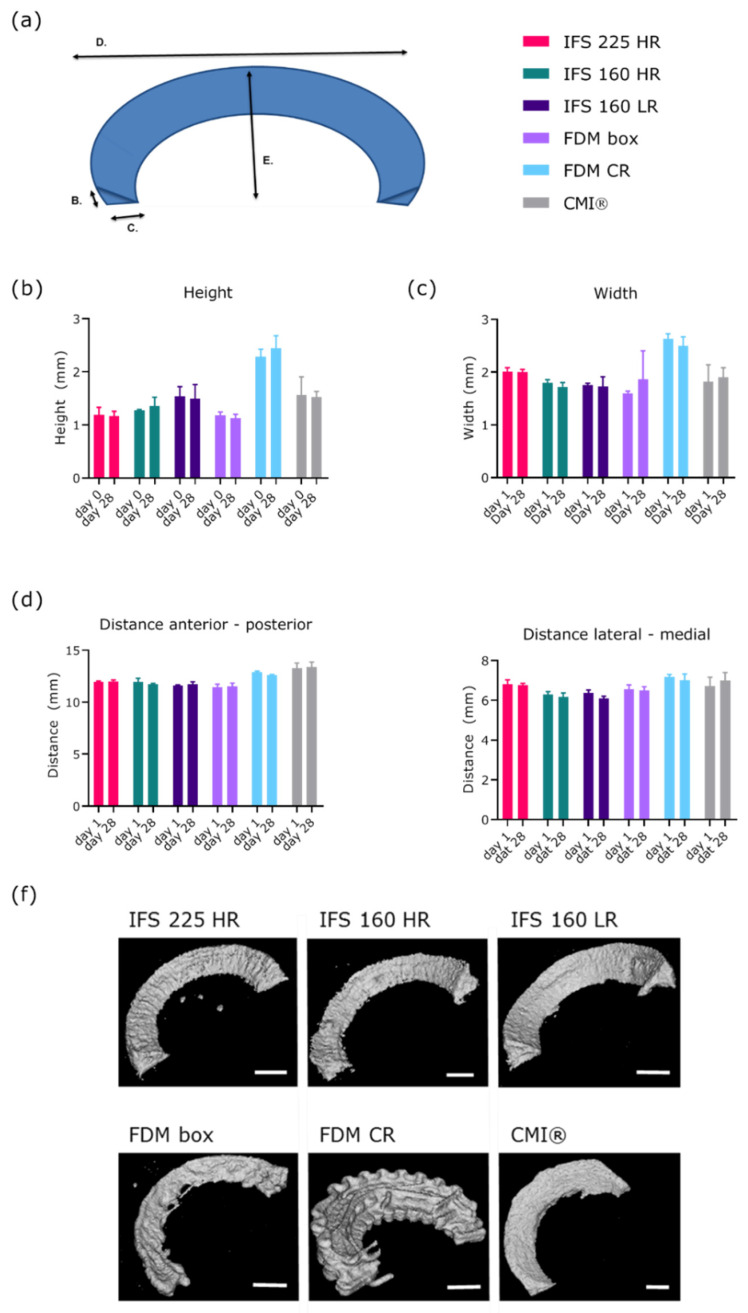
(**a**) Schematic overview of measured dimensions (**b**) height of the scaffold, (**c**) width of the scaffold, (**d**) anterior-posterior distance, (**e**) lateral-medial distance, (**f**) representative µCT images per group after 28 d of culture. *n* = 3 donors, 1-2 technical replicates per donor. Abbreviations; 160: 160 µm, 225: 225 µm, CMI^®^: Collagen Meniscus Implant^®^, IFS: inter fibre spacing, HR: high ratio of radial fibres, LR: low ratio of radial fibres, FDM: fused deposition modelling, CR: circumferential and radial fibres, Box: box-structure. Scale bar is 2 mm.

One of the main functions of the scaffold is to provide a framework and cells might affect the shape of this scaffold during culture. Therefore, shape fidelity was assessed using the dimensions of the scaffolds over the 28-culture period as a measure (Figure 4A). Irrespective of fibre reinforcing tactic or internal fibre structure, scaffolds retained shape over time in height, width, anterior-to-posterior distance, and lateral-to-medial distance (Figure 4B–F). 

### 2.5. Extracellular Matrix Formation During 28 Days of Culture

**Figure 5:**
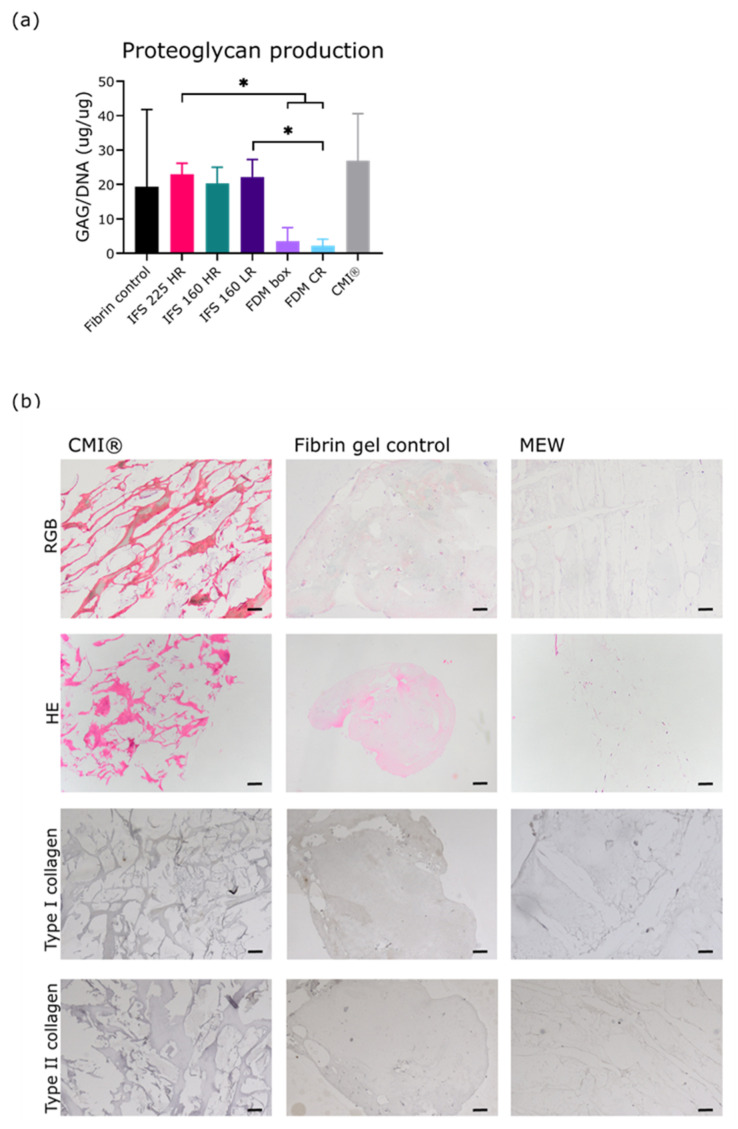
Cell distribution and extracellular matrix formation after 28 d of co-culture. (**a**) GAG production normalized for DNA content of scaffolds (*n* = 3 donors, 3 technical replicates per donor). (**b**) Picrosirius Red, Fast Green and Alcian Blue (RGB), Hematoxylin and Eosin (HE), type I collagen and type II collagen stained sections showing cell distribution and tissue deposition in collagen meniscus implant (CMI), fibrin gel control, and melt electrowriting (MEW) scaffold after 28 d of culture(*n* = 3 donors, 2 technical replicates per donor. *, *p* < 0.05; 160: 160 µm, 225: 225 µm, Box: box-structure, CR: circumferential and radial fibres, FDM: fused deposition modelling, HR: high ratio of radial fibres, IFS: inter fibre spacing, LR: low ratio of radial fibres.

After 28 d of culture, glycosaminoglycan (GAG) production, normalized per DNA, was similar between MEW scaffolds and the fibrin control (Figure 5A). IFS 225 HR had a significantly higher GAG production than both FDM groups (23.0 ± 3.2 vs 3.5 ± 3.9 and 2.2 ± 2.0). GAG production by IFS 160 LR was significantly higher than FDM CR, but not than FDM box (*p* = 0.05). GAG production by IFS 160 HR was not significantly different from FDM groups (*p* = 0.05 and *p* = 0.06). Cells were found throughout the scaffolds on the sections in that were taken at different locations and in two directions (Figure 5B–D). Picrosirius red and Alcian blue staining were observed in fibrin gel controls and MEW scaffolds, but low indicating deposition of small amounts of collagens and proteoglycans. There was minimal deposition of type I collagen in the scaffolds. Collagen type II staining was negative in all scaffolds (Figure 5B).

## 3. Discussion

In this study, a scaled-down meniscus-like scaffold was fabricated from medical grade materials using MEW and seeded with a combination of meniscus cells and MSCs. As the natural architecture of the native meniscus is imperative for its function in load transmission, the fabrication of such a shape was an important aspect of this study. On a macro-scale level, the meniscus consists of a round-rim in the x-y plane and a wedge-shape in the out-of-plane direction. Especially for the MEW scaffolds, this scaled down version did still encompass the macroscopic wedge-shaped structure. With the relatively low resolution of FDM fibre deposition, the wedge shape was less smooth as compared to the MEW scaffolds. The seeded cells remained viable in the scaffold during 28 d culture and produced a basal level of GAGs. During 28 d of culture, the cell-seeded scaffolds increased in yield strength and ultimate strength. MEW scaffolds showed higher strains as compared to FDM scaffolds, suggesting that the MEW scaffolds have a larger elastic region as compared to the FDM ones. 

MEW was used with the aim to replicate the intricate fibre architecture that includes both circumferential and radial orientated fibres. MEW scaffolds can be created from medical grade materials with high precision and reproducibility, which is imperative for clinical translation [25,26,27,28]. To create live-sized scaffolds that reflect natures architecture, the inter fibre distances could be decreased further. The inter fibre spacing achieved in this study (160 and 225 µm) were chosen because of deposition reproducibility in current printing path with the machine used. To improve mechanical properties, the inter fibre spacing could be decreased to increase the overall fibrous content and cell infiltration and migration should then be re-evaluated. A recent study on the limits of inter fibre distances in MEW-based scaffolds reported around 60 µm inter fibre distances, which shows feasibility of decreasing fibre distances [29]. Recently, possibilities in scaffold design are increasing by the fabrication of out-of-plane fibres [30], incorporated spanning fibre sheets [31], and micro-scale layer shifting [32]. The latter uses an offset printing trajectory to overcome the electrostatic autofocussing effect and therefore allows nonlinear geometries [32]. Using an offset printing trajectory, a rounded-rim, wedge shape geometry was made for the first time using MEW technology, which showcases the potential use of MEW for more intricate geometries.In this study, we explicitly chose to use clinical grade materials and cell types and a cell number that can be achieved within a single surgery. The cell density used here was based on the cell concentration used in the treatment of articular cartilage defects [21,33]. This concentration cannot be obtained with autologous meniscus cells without culture expansion [23], therefore a combination of MSCs and fibrochondrocytes [22,34] was used in contrast to previously reported approaches that have used only meniscus cells [35,36]. Although this cell concentration, without the addition of growth factors, does not lead to extensive tissue formation in vitro [22]*,* good results are obtained in vivo using this cell concentration for cartilage defects [21,33] and it is a feasible cell number for use in one-stage treatment. We successfully seeded the scaffolds with this cell combination and showed good cell retention during 28 d culture, which might be attributed to the micro fibre size and small pores. After 28 d of culture, HE staining showed a homogenous distribution of the cells throughout the scaffold. The shape of the scaffolds was stable during the 28 d of culture, although this does not guarantee that the scaffolds will retain their shape in vivo upon mechanical loading. The yield stress and ultimate strength of MEW scaffolds seemed to increase between day 1 and day 28, which indicates tissue formation in the scaffolds. Moreover, formation of a basal level of GAGs (comparable to the fibrin gel control group) was demonstrated. Deposition of collagen and proteoglycans (as indicated by picrosirius red and alcian blue staining) were low in all scaffolds. The aim of this research was to investigate whether any ECM deposition could take place in our scaffolds and compare this to the FDM and CMI^®^ controls. As the aim was not to produce large amounts of extracellular matrix, static culture conditions were used without the supplementation of growth factors. In vivo, the seeded cells will be provided with the stimulating mechanical cues and growth factors in the joint, which might further enhance matrix formation and mechanical properties. Interestingly, FDM scaffolds had a lower GAG production, which could be explained by a lower seeded cell number or the presence of large fibres which both might impair cell communication and EMC production. For clinical translation, the scaffolds should not be subject to fast resorption in vivo. PCL fibres are still present 6 months after implantation in and equine joint after extensive loading. This suggests suitability of the PCL scaffolds for clinical usage [37].

### 3.1. Limitations

This study shows that it is feasible to obtain similar compressive properties with medical grade cell-laden materials and microscale MEW fibres as compared to the CMI^®^. Although promising, it should be noted that a scaled down model of the meniscus rather than a full-size meniscus was used here in order to enable high throughput screening in vitro. Even though the fabrication of live-sized scaffolds for clinical use should be possible [38], the mechanical properties of such a full-scale scaffold should be re-evaluated. Additionally, the compressive properties of these scaffolds are not within the range of human native meniscus yet, as native meniscus has a Young’s modulus in the megapascal range [39]. However, the improvement in yield strength and ultimate stress within 28 d of static culture demonstrate the potential of this approach using cells. In the current approach for mechanical testing, fibres undergo tensile forces while stretching from compressive loading. However, this does not include compression under different angles of sliding motion, or pull-out testing. By using a custom-made compression head, the complete wedge underwent compression. Surface roughness was not assessed in this study, but previous in vivo studies using fibres of comparable thickness deposited using MEW showed no damage to the opposing structures in the joint ([37]). Lastly, overall tissue formation was limited in this study, which might be attributed to the low cell numbers, static culture conditions and absence of growth factor stimulation. In the current approach, we explicitly choose to use clinically feasible cell numbers in order to facilitate clinical translation as one-stage treatment, in which tissue formation by the seeded cells will be guided by the joint environment after implantation. Nonetheless, we did not compare formation of more meniscus specific extracellular matrix (e.g., type I collagen), as immunohistochemistry is not sensitive enough for such small amounts of formed tissue. Instead, we used GAG production to assess tissue formation, which is commonly used in meniscus research even though the GAG content in an healthy meniscus is generally relatively low [40]. 

### 3.2. Implications

This study demonstrates feasibility of creating wedge-shaped MEW scaffolds seeded with clinically feasible cell numbers and types for potential translation as one-stage treatment. The efficacy of these scaffolds for meniscus replacement should be further evaluated in vivo. 

## 4. Materials and Methods

### 4.1. Scaffold Design and Printing

**Figure 6:**
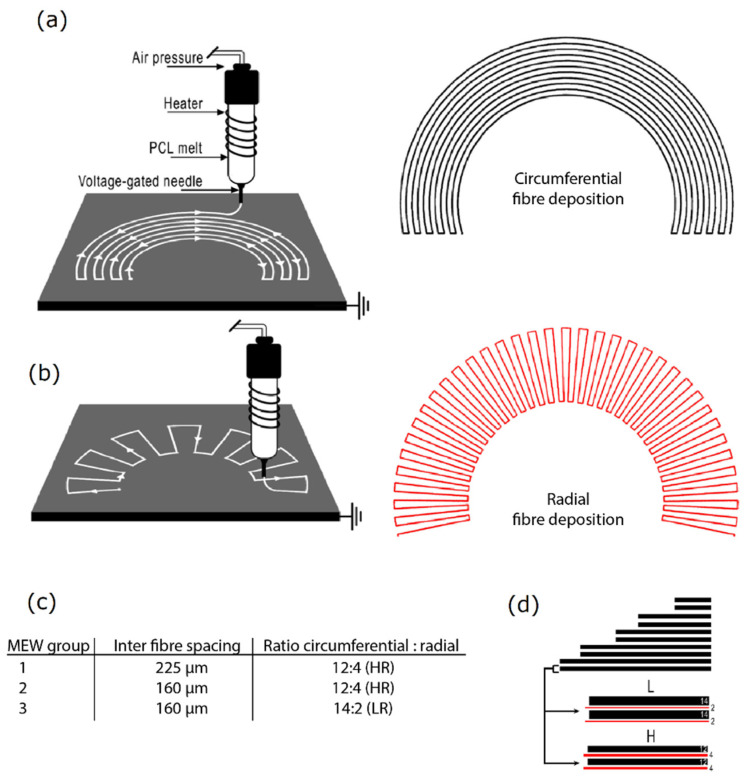
Scaffold design inspired by native fibre architecture. (**a**) Printhead trajectory of circumferential fibres. (**b**) Printhead trajectory of radial fibres. (**c**) Variables in design include variety in inter fibre spacing and in the ratio between the circumferential and radial fibres. (**d**) Illustration of variety in design of the ratio of circumferential and radial fibres. HR: high ratio of radial fibres, LR: low ratio of radial fibres, PCL: polycaprolactone.

Scaffold design was based on native meniscus fibre architecture using micro-meter scale fibres in a circumferential (Figure 6A) and radial (Figure 6B) direction using MEW. These two different layers were deposited with a programmed inter fibre spacing of 225 μm or 160 μm (Figure 6C) and the ratio of circumferential: radial fibres was 14:2 (low radial, LR) or 12:4 (high radial, HR) (Figure 6C,D). As for high throughput testing, the meniscus scaffolds were scaled down by a factor of 4 to fit into 24 well culture plates.

MEW was performed with polycaprolactone (PCL, PURASORB, Corbion, Gorinchem, The Netherlands) at 90 °C, a collector distance of 5 mm, collector velocity of 10 mm/s, voltage of 9 kV, at a pressure of 0,118 MPa (3D Discovery, regenHU, Villaz-Saint-Pierre, Switzerland). Printability was assessed by measuring the fibre diameter and inter fibre spacing along the circumferential and radial lengths of the prints. These measurements were performed on images taken with scanning electron microscope (SEM, Phenom Pro Desktop SEM, Thermo Fisher Scientific, Waltham, MA, USA) by using Fiji software (ImageJ, version 2.0.0-rc-54/1.51 h). SEM was performed with an accelerating voltage of 10 kV to image the MEW fibres. Prior to imaging, samples were coated with 2 nm of gold to improve imaging quality. Homogeneity of the fibre diameter was assessed by the standard deviation and the measured inter fibre spacing was compared to the programmed inter fibre spacing. To assess if the ratio of circumferential: radial fibres was achieved, SEM imaging was used with the same parameters as for the fibre measurements. FDM scaffolds were made from PCL with screw-driven extrusion at 3 rev/min, an air pressure of 0.125 MPa, and a collector velocity of 2 mm/s at a temperature of 80 °C (3D Discovery, regenHU). 

### 4.2. Cell Isolation and Culturing

Primary human meniscus cells were isolated from osteoarthritic menisci obtained after total knee arthroplasty from 3 female donors (62–81 years old). The tissue was handled anonymously according to the guidelines of the Federation of Dutch Medical Scientific Societies[41] and as approved by the ethical review board of the University Medical Center Utrecht. Briefly, menisci were cut into 1–2 mm cubical pieces and digested in Dulbecco’s modified Eagle’s medium (DMEM; Gibco, Life Technologies Europe B.V., Bleiswijk, the Netherlands) with 0.2% pronase (Roche Diagnostics GmbH, Mannheim, Germany), 100 U/ml penicillin (Gibco) and 100 µg/mL streptomycin (Gibco) (1% p/s) at 37 °C for 2 h followed by a digestion in DMEM with 0.075% collagenase type 2 (Wortington Biochemical Corporation, Lakewood, NJ, USA), 1% p/s, and 10% heat-inactivated fetal bovine serum (FBS; Biowest, Nuaillé, France) at 37 °C. The digested tissue was run over a 70 µm strainer (Greiner Bio-One International GmbH, Kremsmünster, Austria) to remove debris, after which meniscus cells were cultured up to passage 2 in DMEM with 1% p/s and 10% FBS.

The use of human MSCs was approved by the institutional ethical review board (TCBio 08-001 and 18/739). MSCs were obtained from bone marrow aspirates from 4 patients (male and female, age 35-71) undergoing hip replacement or spinal surgery after written informed consent was obtained. Briefly, bone marrow aspirate was Ficoll separated and MSCs were expanded up to passage 4-5 in αMEM (minimal essential medium, Gibco) with 10% FBS, 1% 20 mM L-ascorbic acid-2-phospate (1% ASAP; Sigma-Aldrich, Saint Louis, MO, USA) and 1% p/s. 

### 4.3. Scaffold and CMI^®^ Preparation

CMIs^®^ were reduced to the same dimensions of the MEW scaffolds using a cutting guide. Prior to seeding the downscaled CMIs^®^ with cells, they were treated with 1% p/s and 50µg/ml gentamicin in PBS for 7 d and dried overnight. Scaffolds were treated with 1 M NaOH in H_2_O to increase hydrophilicity and improve immersion of the scaffolds with fibrin gel. 

### 4.4. Seeding of The Scaffolds

Tisseel fibrin gel (Tisseel, Baxter BV, Utrecht, The Netherlands) was used in a 1:50 dilution of the thrombin component (= 10 IU thrombin/mL with 8 µmol/mL calcium chloride) and a 1:15 dilution of the fibrinogen component (= 5–8 mg fibrinogen, 1-3 IU/mL factor XIII with 20o KIU/mL aprotinin). Interconnectivity of the fibrin glue fibers [42] was not measured in the current study. MSCs and meniscus cells were mixed in a 20:80 ratio in the of fibrinogen in PBS. The CMIs^®^ and MEW scaffolds were placed in a seeding mold, after which 30 µL fibrinogen solution containing a total of 1.5 × 10^5^ cells was added. Thrombin was added and the fibrin gel was allowed to gelate for 20 min at 37 °C. The seeded scaffolds were cultured at 37 °C/5% CO_2_ for 28 d in DMEM with 1% p/s, 2% Albuman (human serum albumin, 200 g/L; Sanquin Blood Supply Foundation, Amsterdam, the Netherlands), 2% insulin-transferrin-selenium-ethanolamine (ITS-X; Gibco), and 1% ASAP, with medium changes twice a week. Low attachment plates suspension plates (Greiner Bio-One) were used to prevent attachment of released cells to the bottom. Therefore, the amount of DNA released in the culture medium could be used as a measure of cell retention in the scaffold. DNA content after seeding and DNA release in the first week after seeding was quantified using the Quant-iT PicoGreen kit (Invitrogen, Invitrogen, Carlsbad, CA, USA) according to the manufacturer’s protocol, with excitation at 485 nm and emission at 535 using the Fluoroskan Ascent (Thermo Fisher Scientific) for three technical replicates (one donor combination). Cell distribution in the scaffold was visualized on a thunder microscope (Leica microsystems, Wetzlar, Germany) after staining the cells with 10 µM calcein-AM (Thermo Fisher Scientific) for 30 min at 37 °C. 

### 4.5. Mechanical Analysis

**Figure A1:**
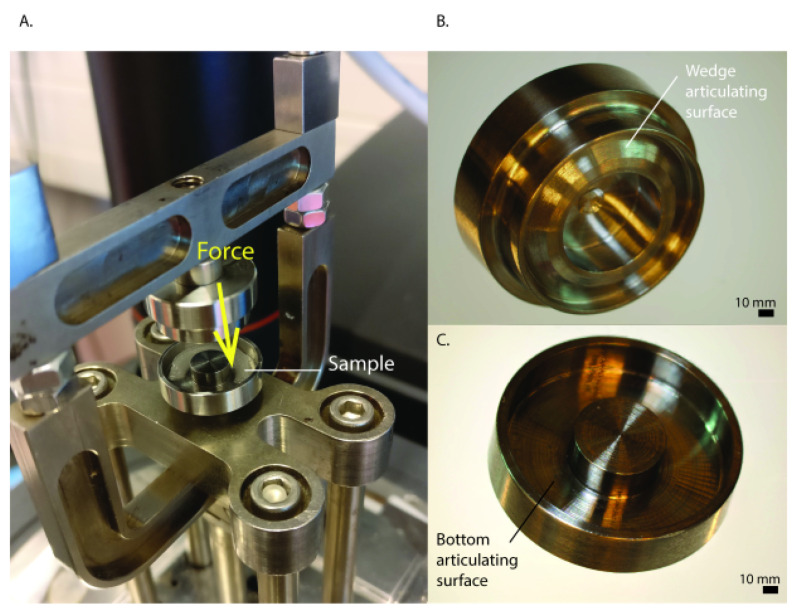
Mechanical characterization of scaffolds. (**A**) Compressive testing system, (**B)** Custom made scaffold holder for compression of the wedge surface (top) and (**C**) Custom made scaffold holder for compression (bottom).

**Figure A2:**
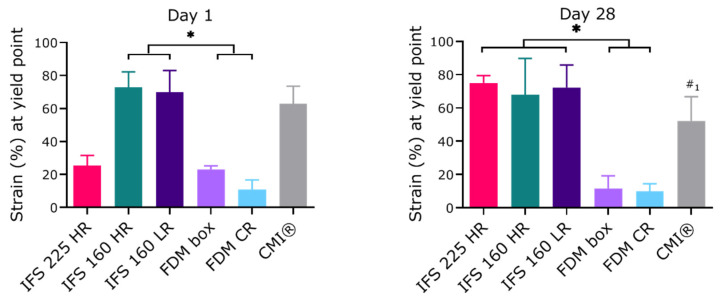
Strain (%) at yield point for all scaffolds at day 1 and day 28. *, *p* < 0.05; #_1_ *p* < 0.05 compared to all groups except IFS 160 HR. Abbreviations; 160: 160 µm, 225: 225 µm, CMI^®^: Collagen Meniscus Implant^®^, IFS: inter fibre spacing, HR: high ratio of radial fibres, LR: low ratio of radial fibres, FDM: fused deposition modelling, CR: circumferential and radial fibres, Box: box-structure.

The mechanical properties were analysed using confined compression of the scaffolds with an aluminium custom-made loading head in the shape of the scaffolds on a Dynamical Mechanical Analyser (DMA, Q800, T.A. Instruments, New Castle, DE, USA) (Appendix A, Figure A1). A preload of 0.001 N was applied after which the scaffolds were compressed until 30% of the original height with 20% compression per minute. Compressive Young’s modulus was calculated from the slope of the stress-strain curves. To determine the yield point, yield strength and ultimate strength, a force ramp of 1.5 N/min to 18 N was performed. Mechanical properties were assessed for five technical replicates (one donor combination) at day 1 and three technical replicates per donor combination (three donor combinations) at day 28. 

### 4.6. Computed Tomography

To analyse scaffold shape after seeding and culture, scaffolds were imaged through µCT scanning using a Quantum FX µCT scanner (voxel size = 29.29 µm^3^ µm^3^, 90 kV tube voltage, 200 µA tube current, and 26 s of scan time, Perkin Elmer, Waltham, MA, USA) after 1 and 28 d of culture. Using ImageJ, three dimensional images were assembled and the landmarks function was used to measure the scaffolds. 

### 4.7. Extracellular Matrix Formation

After 28 d of culture, scaffolds were digested at 60 °C overnight in papain solution (50 μg/mL papain; Sigma-Aldrich, 0.2 M NaH_2_PO_4_, 0.1 M EDTA, 0.01 M cysteine, pH 6). Proteoglycan content of the scaffold and proteoglycan release into the culture medium were assessed using the dimethylmethylene blue (DMMB; pH 3) assay to quantify sulphated GAGs. Chondroitin-6-sulfate (Sigma-Aldrich) was used as a standard. Absorbance was measured at 525 and 596 nm. Proteoglycan production was normalized for DNA, which was quantified as indicated above. Matrix formation was assessed for three technical replicates per donor combination (three donor combinations). 

### 4.8. Histology

Scaffolds were fixed and embedded in paraffin in two orientations and cut into 5 µm sections. Cell morphology and distribution were assessed using Haematoxylin and Eosin staining and RGB staining [43]. For RGB staining, sections were stained with 1% Alcian blue in 3% aqueous acetic acid (pH 2.5) for 20 min and rinsed in tap water. Following this, sections were stained with 0.04% fast green in distilled water for 20 min and rinsed in tap water for 5 minutes. Lastly, sections were stained with 0.1% Picrosirius red for 30 min, followed by 2 changes of 5 min in 1% acidic acid in tap water. For type I and II collagen immunohistochemistry, antigen were retrieved using 1 mg/mL pronase (Sigma-Aldrich) followed by blocking with 5% bovine serum albumin (BSA) in PBS for 30 min. Samples were incubated with the primary antibody (type I collagen, rabbit monoclonal 1/400 in PBS/BSA 5% or type II collagen, mouse monoclonal 1/100 in PBS/BSA 5%) overnight. Sections were incubated with horseradish peroxidase conjugated anti-rabbit secondary antibody (DAKO, Glostrup, Denmark) for 30 min after washing. Immunoreactivity was visualized using diaminobenzidine peroxidase substrate solution (DAB, Sigma-Aldrich) and sections were counterstained with Mayer’s hematoxylin.

### 4.9. Statistics

Data were analyzed using GraphPad Prism version 8.3 (GraphPad Software, San Diego, CA, USA). Data are shown as mean ± standard deviation (SD). A student’s *t*-test was used to compare measured inter fibre spacing. Young’s modulus and proteoglycan production was compared between MEW groups and all other groups using Welch ANOVA with a Dunnett’s T3 correction for multiple comparisons since variances were not equal. Similarly, the DNA content was compared between the fibrin control and different scaffolds using Welch ANOVA with a Dunnett’s T3 correction. DNA release in medium was regarded illustrative data and no statistics were performed on this data. Yield points, yield strength and ultimate strength were compared using ordinary ANOVA with a Sidak correction. Assumptions were checked visually using residual, homoscedasticity and QQ plots. *p*-values below 0.05 were assumed significant and indicated by *. 

## Data Availability

The data presented in this study are available on request from the corresponding author.
